# Vaccine Adjuvants Differentially Affect Kinetics of Antibody and Germinal Center Responses

**DOI:** 10.3389/fimmu.2020.579761

**Published:** 2020-09-23

**Authors:** Gabriel Kristian Pedersen, Katharina Wørzner, Peter Andersen, Dennis Christensen

**Affiliations:** ^1^Center for Vaccine Research, Statens Serum Institut, Copenhagen, Denmark; ^2^Department of Immunology and Microbiology, University of Copenhagen, Copenhagen, Denmark

**Keywords:** adjuvant, vaccine, antibody, kinetics, germinal center (GC), alum, squalene emulsion, CAF01

## Abstract

Aluminum salts and squalene based oil-in-water emulsions (SE) are widely used adjuvants in licensed vaccines, yet their mechanisms are not fully known. Here we report that induction of antibody responses displays different kinetics dependent on the adjuvant used. SE facilitated a rapid antibody response in contrast to aluminum hydroxide (AH) and the depot-forming cationic liposome-based adjuvant (CAF01). Antigen given with the SE adjuvant rapidly reached follicular B cells in the draining lymph node, whereas antigen formulated in AH or CAF01 remained at the site of injection as a depot. Removal of the injection site early after immunization abrogated antibody responses only when antigen was given in the depot-forming adjuvants. Despite initial delays in B cell activation and germinal center (GC) formation when antigen was given in depot-forming adjuvants, the antibody levels reached higher magnitudes than when the antigen was formulated in SE. This study demonstrates that the kinetic aspect of antibody responses is critical in adjuvant benchmarking and suggests that the optimal vaccination regime depends on the adjuvant used.

## Introduction

Antibody responses are the best correlate of protection against many infectious diseases and vaccines inducing optimal antibody responses are therefore desired (1). Adjuvants are used to augment or modify immune responses and the choice of adjuvant can impact both the affinity, specificity, and functional profile of the elicited antibody response (2, 3). The mechanism of action for adjuvants is becoming increasingly well-defined, particularly for those that ligate pathogen recognition receptors, such as toll-like receptors (4). However, for many adjuvants, such as Aluminum-based (e.g., Aluminum hydroxide (AH), often referred to as “Alum”) and squalene based oil-in-water emulsions (e.g., MF59™), the mechanism of action is less clear and may be a combinational effect [e.g., induction of apoptosis and DAMP signaling (4–6)]. Some adjuvants also retain antigen at the site of injection and the ability of aluminum salts to induce an antigen depot was long believed to be essential for the adjuvant effect, although this has lately been questioned (7, 8). With an increasing number of adjuvants going from pre-clinical to clinical development, there is an unmet need for adjuvant benchmarking studies (9).

Optimal elicitation of antibody responses requires a number of ordered events. Priming of antibody responses against T-dependent antigens predominantly occurs in lymph nodes (LN) draining the site of injection (10) upon acquisition of antigen by follicular B cells (11). After interacting with T helper cells at the T/B border, responding B cell clones can initiate a germinal center (GC) reaction, in which the B cells proliferate, mature their B cell receptors to increase affinity and perform class-switching (12). A prerequisite to GC formation is transport of unprocessed antigen to lymphoid organs, which may occur *via* active cellular transport or lymphatic drainage (13, 14). Strategies to promote transport to follicular B cells, such as antigen acquisition by innate immune cells at the site of injection (15), their migration to LNs and delivery of antigen to follicular dendritic cells and B cells (16), therefore hold the potential to favor germinal center reactions, induce class-switching and promote high-affinity antibody responses. On the contrary, adjuvants that sequester antigen at the site of injection may cause a limited antigen presentation to follicular dendritic cells and B cells, which could manifest as reduced or delayed B cell activation, germinal center formation and resulting antibody responses. Notably, slow-release of antigen could also be of advantage, since a constant feeding of antigen to existing germinal centers may aid in sustaining these, possibly promoting affinity maturation (17–19). Data supporting this theory have been generated using continuous antigen delivery either by repeated boosting, mini-pellets, microneedles or *via* osmotic sustained release pumps (17, 20–23). However, studies addressing how vaccine depot formation influences immune response kinetics are lacking.

In previous studies comparing clinically tested adjuvants, emulsion-based adjuvants (e.g., MF59^TM^ and GLA-SE) promoted a rapid increase in antibody titers, whilst adjuvants which have been suggested to form a vaccine depot [e.g., CAF01 and AH (5, 24), gave distinctly low responses early after immunization (9, 25–27)]. We hypothesized that antibody response kinetics depend on the type of adjuvant applied, and since these studies investigated responses only 7–14 days post immunization, it was possible that the adaptive immunity had not fully matured at these early time points. We therefore performed a study to follow kinetics of GC formation and antibody responses after a single immunization with antigen formulated in adjuvants. We compared adjuvants reported to retain antigen at the site of injection [CAF01 (24, 28) and AH (5)], to emulsions [MF59™-like squalene emulsion AddaVax™ (SE)] that are reported not to retain antigen (29). Antigen tracking studies revealed that CAF01 and AH indeed facilitated retention of antigen at the site of injection, whilst antigen formulated in SE rapidly associated with follicular B cells in draining LNs. Moreover, we found that GC's appeared earlier in mice immunized with antigen formulated in SE adjuvant than with the CAF01 and AH adjuvants and that this was paralleled by a faster antibody response. However, at later time points the depot-forming adjuvants resulted in higher magnitude antibody responses than the non-depot-forming adjuvant. This study shows that adjuvants differentially affect GC kinetics, which may influence the optimal timing for booster vaccinations and is essential to take in to account when comparing different adjuvants.

## Materials and Methods

### Mice

Female CB6F1 (CB6F1/OlaHsD) wild type mice, 6–8 weeks old, were ordered from Harlan Laboratories (The Netherlands) and housed in the animal facilities at Statens Serum Institut, Denmark. Mouse studies were conducted in accordance with the regulations set forth by the national animal protection committee and in accordance with European Community Directive 86/609. The experiments performed have been approved by the governmental Animal Experiments Inspectorate under licenses 2014-15-2934-01065 and 2017-15-0201-01363.

### Antigens and Adjuvants

*Chlamydia trachomatis* antigen CTH522 (MOMPextVD4) and *M. tuberculosis* H56 antigens were recombinantly produced in *E. coli* K12 as described previously (9, 30). OVA-AF647 was from Invitrogen and NP_20_-OVA (4-Hydroxy-3-nitrophenylacetyl-OVA) from Biosearch technologies. CAF01 (DDA/TDB) was produced in house (Statens Serum Institut, Copenhagen, Denmark) (31), The AddaVax^TM^ oil-in-water squalene emulsion (SE) was from Invivogen (Toulouse, France) and aluminum oxyhydroxide (AH) (2% Alhydrogel®) was from Brenntag Biosector (Frederikssund, Denmark).

### Immunizations

Mice were given a single immunization subcutaneously (s.c.) at the base of the tail with 5 μg recombinant CTH522 antigen (SSI) or NP-OVA (Biosearch Technologies) in a volume of 200 μl TRIS buffer (pH 7.4) per immunization. Adjuvant doses were according to manufacturer's instructions: CAF01 (dose 250 μg/50 μg (DDA/TDB), SE (dose of 100 μl 4.3% w/v squalene, 0.5% w/v Tween 80, 0.5% w/v Span 85 mixed 1:1 with antigen/PBS) and AH (dose of 500 μg Aluminum content). Control mice (antigen alone) received 5 μg recombinant antigen in 200 μL PBS.

### Surgery for Injection Site Removal

Mice were injected intradermally (i.d.) at the back (after cutting the hair with electric clippers) with CTH522 (5 μg) in CAF01 (125 μg DDA/25 μg TDB), SE (mixed 1:1 with antigen/PBS) or AH (dose of 125–250 μg Aluminum content) in a total volume of 25–50 μl. Antigen depots were removed at various time points after vaccine administration. Mice were anesthetized using Zoletil-mix (tiletaminhydrochloride/zolazepamhydrochloride/xylazin/butorphanol) and a small incision was made in the skin to remove the antigen depot. The incision was closed with tissue adhesive (3M Vetbond) and/or surgical silk-thread (Vicryl 6-9, Ethicon) and Carprofen analgesia was administered for 4 days post-surgery. All mice, including the control group, were anesthetized and received analgesic treatment.

### Organ Preparation

LNs and spleens were filtered through a 70 μm nylon mesh (BD Biosciences). Muscle tissue was treated with enzymes A, D, and P of the Skeletal Muscle Dissociation Kit (Miltenyi Biotec GmbH, Bergisch Gladbach, Germany) according to the manufacturer's instructions. Muscle tissues were then prepared for single cell suspensions using the GentleMACS system (Miltenyi Biotec GmbH, Bergisch Gladbach, Germany). Muscle supernatants were used for cytokine profiling (see below). The cells were washed and prepared as previously described (9) and re-suspended in cell culture medium (RPMI-1640 supplemented with 5 × 10-^5^ M 2-mercaptoethanol, 1% pyruvate, 1% HEPES, 1% (v/v) premixed penicillin-streptomycin solution (Invitrogen Life Technologies), 1 mM glutamine, and 10% (v/v) fetal calve serum (FCS). The cells were adjusted to 2 × 10^5^ cells/well (MSD/ cytokine ELISA) or 1–2 × 10^6^ cells/well (Flow cytometry).

### *In vivo* Tracking Studies

OVA coupled to AlexaFluor (AF) 647 (Thermo Scientific) was mixed with TRIS buffer (pH 7.4) and administered alone or with the indicated adjuvant at a dose of 5 μg either intramuscularly (i.m.) (50 μl) in the thigh muscle or s.c. (200 μl) at the base of tail as stated. Mice were euthanized 6, 24, 48 h or 7 days after the injection. Muscle tissue and draining LNs (inguinal) were isolated and used for flow cytometry.

### Cytokine Profiling

The Mouse U-plex (custom cytokine: MIP-1α, IL-12p70, IL-1β, IL-6, TNF-α, MCP-1, IL-5, and IL-10) was performed according to the manufacturer's instructions (Meso Scale Discovery) to measure cytokine concentrations in muscle supernatants. The plates were read on the Sector Imager 2400 system (Meso Scale Discovery) and calculation of cytokine concentrations in unknown samples was determined by 4-parameter logistic non-linear regression analysis of the standard curve. IFN-γ and IL-17 responses were measured by ELISA as described previously, using supernatants from splenocyte cultures stimulated *in vitro* with CTH522 antigen (2 μg/ml) in cell culture medium for 72 h at 37°C and 5% CO_2_ (30).

### Immunohistology

Mice were injected into the thigh muscle with CTH522 alone or in the presence of the indicated adjuvant. At 6, 24, and 48 h and at 7 and 60 days post injection, muscles were collected and fixed in formalin. Tissues were embedded into paraffin and sectioned to 4 μm thickness. Hematoxylin (HE) (Histolab Ab) and Immunoperoxidase staining followed by rabbit anti-CD64 (Sino Biologicals) and HRP-polymer anti-rabbit antibody (Nordic Biosite) was performed. Tissues were scored for CD64 positive cells as <100 (0), 100–1,000 (1), 1,000–2,000 (2), 2,000–5,000 (3) or >5,000 cells (4) per muscle.

Inguinal LNs were isolated following subcutaneous injection at the base of tail with CTH522 alone or in the presence of the indicated adjuvant and at various time points after administration assessed for germinal centers. 4 μm thick paraffin-embedded tissue sections were stained with HE and rabbit anti-Ki67 (Sp6) followed by HRP-polymer anti-rabbit antibody (Nordic Biosite). Germinal centers were identified as clusters of Ki67 positive cells and the surface areas of the germinal centers were measured. Slide quality was controlled utilizing an Olympus BX-60 microscope and an integrated Scion color digital camera. Slides were digitalized utilizing a 3D-Histech Panoramic MIDI and HV-F22 Hitatchi camera and interpreted with Case Viewer software. Stainings and interpretation of slides were assessed by a pathologist who was blinded to the treatment groups.

### ELISA for Antibody Responses

Maxisorb Plates (Nunc) were coated with 0.05 μg/well CTH522 overnight at 4°C. Individual mouse sera were analyzed in duplicate. After blocking, serum was added in PBS with 2% BSA, starting with a 30-fold dilution for antigen-specific IgM, IgG or IgG subclasses. HRP-conjugated secondary antibody [rabbit anti-mouse IgG (Zymed), Goat anti-mouse IgG1 (Southern Biotech) or IgG2c (Thermofischer)] was diluted in PBS with 1% BSA. For detection of IgM, serum was added in PBS with 5% skimmed milk and detection was done using biotin conjugated anti-mouse IgM (Southern Biotech) for 1 h followed by streptavidin-HRP (BD Biosciences). After 1 h of incubation, antigen-specific antibodies were detected using TMB substrate as described by the manufacturer (Kem-En-Tec Diagnostics). The absorbance values were plotted as a function of the reciprocal dilution of serum samples. Antibody titers were determined as the highest serum dilution corresponding to a cut-off of ≥0.2 OD450. To measure anti-NP antibody responses, ELISA plates were coated with 0.1 μg/well of BSA coupled with different ratios of NP (NP2-BSA and NP13-BSA) (Biosearch Technologies).

### Flow Cytometry

One million cells were stained in PBS+1% FBS. Cocktails of antibodies against the following surface proteins were used: B220 PerCP-Cy5.5 (RA3-6B2), B220 FITC (RA3-6B2), GL7 BV421 (GL7), IgD BV786 (11-26c.2a), Ly6G PE (1A8), CD11b PerCP-Cy5.5 (M1/70), CD11b PE-Cy7 (M1/70), CD4 APC (RM4-5), CxCR5 BV421 (2G8), CD11c BV421 (HL3) (All BD) CD38 PE-Cy7 (90), F4/80 PE-Cy7 (BM8), F4/80 APC-EF780 (eBioscience), CD11c APC-Cy7 (N418), and PD-1 BV605 (29F.1A12) (Biolegend). A live/dead marker was used to exclude dead cells in the GC B and TFH cell panels (Fixable Viability Dye eFluor™ 780, eBioscience). AF647-labeled ovalbumin (OVA-AF647) was from Invitrogen. Antigen-specific germinal center B cells were measured by including CTH522 coupled to AF488 as probe (conjugated by Life technologies at a coupling ratio of 3 moles dye/mole). Cells were analyzed on a BD Fortessa or FACSCanto flow cytometer.

### Statistical Analysis

Differences between adjuvanted groups were analyzed by Kruskal-Wallis test (antibody titres), using the SE group as reference, and Dunn's test for multiple comparisons or One-way ANOVA, using the SE group as reference, and Dunnett's test for multiple comparisons. Prism 8 software (GraphPad v8.2.1) was used for all statistical analyses.

## Results

### Adjuvants Differentially Influence Kinetics of Antibody Responses

**Figure 1:**
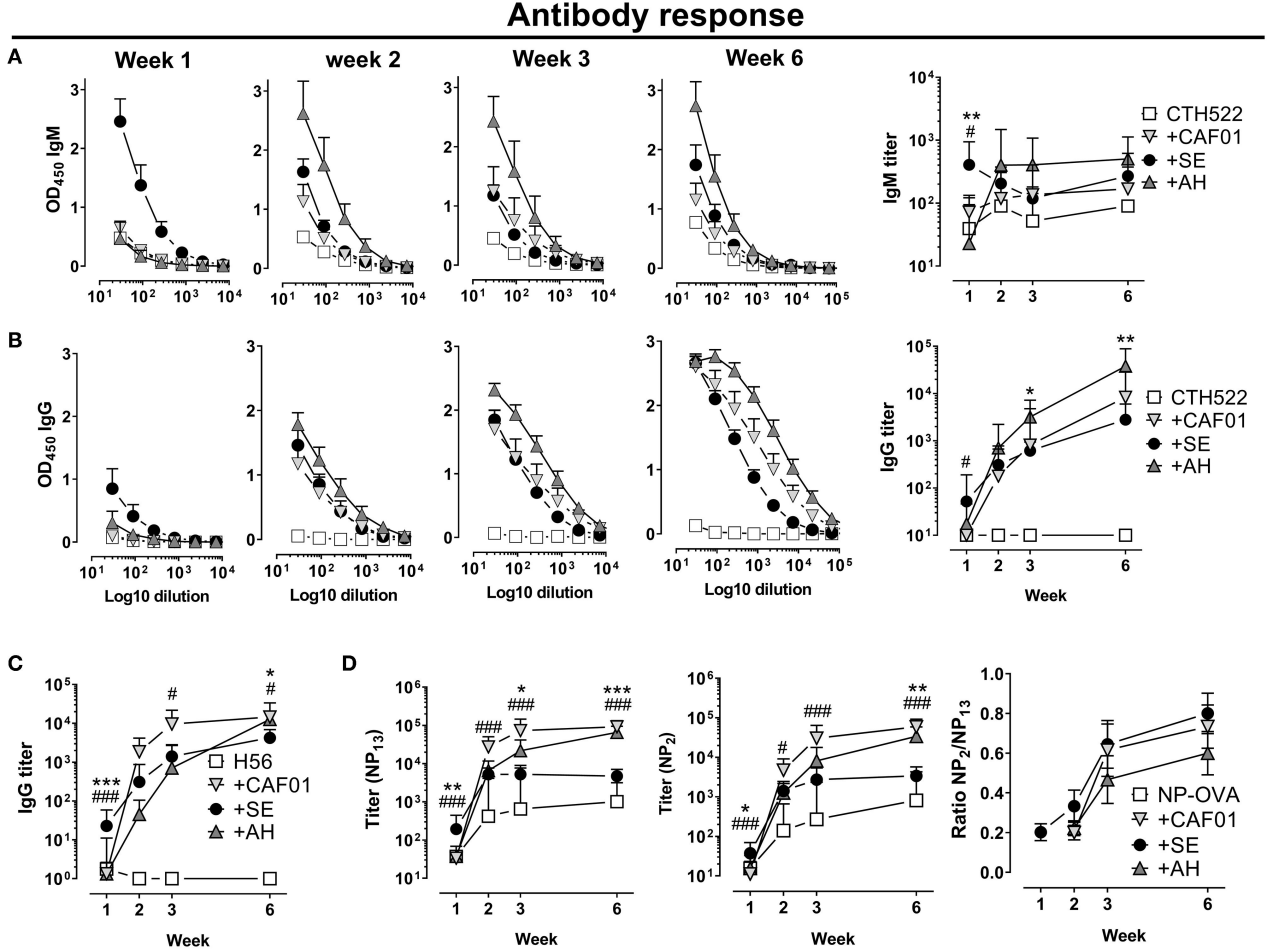
Distinct kinetics of antibody responses dependent on the adjuvant. Mice were vaccinated subcutaneously with 5 μg of the recombinant protein antigen **(A,B)** CTH522 or **(C)** H56 either alone or in the presence of CAF01, SE (squalene emulsion), or AH (aluminum hydroxide). Antigen-specific IgG antibody responses were measured at the indicated time points. **(C)** Antibody response kinetics to immunization with NP-OVA. Mice were immunized s.c. with NP-OVA (5 μg) alone or in the presence of the indicated adjuvant and total and high affinity NP-specific IgG antibodies were measured by coating with NP_13_-BSA or NP_2_-BSA, respectively. Data show titers expressed as geometric mean+95% CI or ratios of NP_2_/NP_13_ titers+SEM of four mice in the non-adjuvanted groups or eight **(A–C)** to 10 **(D)** mice in the adjuvanted groups. Statistically significant differences between the SE and AH groups are indicated by *, **, and ***, whilst significant differences between the SE and CAF01 groups are indicated by ^#^ and ^###^ (Kruskal Wallis test, using the SE group as reference and significance levels of *p* < 0.05 and *p* < 0.001, respectively). Data are representative of one **(C)** or two **(A,B,D)** experiments.

To investigate kinetics of antibody responses, we performed subcutaneous immunizations with the clinically tested *Chlamydia trachomatis* protein antigen CTH522 (32) formulated in either CAF01, SE or AH and analyzed antigen-specific IgM and IgG antibody responses. SE facilitated a rapid increase in IgM antibody at 7 days following immunization (significantly higher than in the CAF01 (*p* = 0.041) and AH (*p* = 0.003) groups, whilst in the AH and CAF01 groups IgM responses remained low until day 14 (Figure 1A). Antigen-specific IgG responses were also highest in the SE group at day 7 [Significant compared to the CAF01 group (*p* = 0.014) (Figure 1B)], whilst at day 14 IgG responses were similar in all groups. At days 21 and 42 IgG titers were higher in mice that had received AH (significant, *p* = 0.002–0.031) and CAF01 (not significant *p* = 0.34) as compared to the SE adjuvant. The IgG responses consisted predominantly of IgG1 in all adjuvanted groups, whereas IgG2c responses were low after a single immunization (Supplementary Figure 1). Similar kinetics of antibody responses were seen when using the *Mycobacterium tuberculosis* fusion protein H56 (33) (Figure 1C), for which SE facilitated significantly higher responses at 7 days post immunization than CAF01 and AH (*p* = 0.0007), whilst the opposite was found at day 42, where both CAF01 and AH performed better than the SE group (*p* = 0.014–0.026).

To measure how the different adjuvants affected affinity maturation, we immunized mice with the model-antigen NP-OVA and measured circulating total NP-binding (NP_13_-binding) and high affinity (NP_2_-binding) IgG antibodies at various time points (34). Early after immunization (day 7), significantly higher total NP-binding antibody responses were found in the SE group compared to in the CAF01 (*p* = 0.0009) and AH (*p* = 0.003) groups (Figure 1D). However, whilst circulating anti-NP antibody levels plateaued in mice that had received SE adjuvanted vaccine at days 21 and 42 post immunization, responses continued to increase in those that had received CAF01 or AH. Thus, at day 42 post immunization, responses were 10–20 fold higher in the CAF01 (*p* < 0.0001) and AH (*p* = 0.0004) groups compared to the SE group. Similarly, high-affinity (NP_2_-binding) antibody titers were significantly higher in the SE group than in both the CAF01 (*p* = 0.0006) and AH (*p* = 0.032) groups at day 7 post immunization (Figure 1D), whereas the CAF01 and AH induced high-affinity IgG titers reached significantly higher levels than in the SE group at day 42 (*p* = 0.0001–0.002). Despite the higher titers of high affinity (NP_2_-binding) antibodies in the CAF01 and AH groups, the relative binding affinity, as indicated by the ratio of NP_2_-binding and NP_13_-binding antibodies, was similar in all the adjuvanted groups. Overall, these data demonstrate that adjuvants differentially influence the kinetics of antibody induction both qualitatively and quantitatively. We hypothesized that the delayed antibody response observed when antigen was formulated in the AH and CAF01 adjuvants was due to impaired antigen transport to LNs and therefore studied how the different adjuvants affected antigen retention at the site of injection and lymph node drainage.

### CAF01 and AH Retain Antigen at the Site-of-Injection

**Figure 2:**
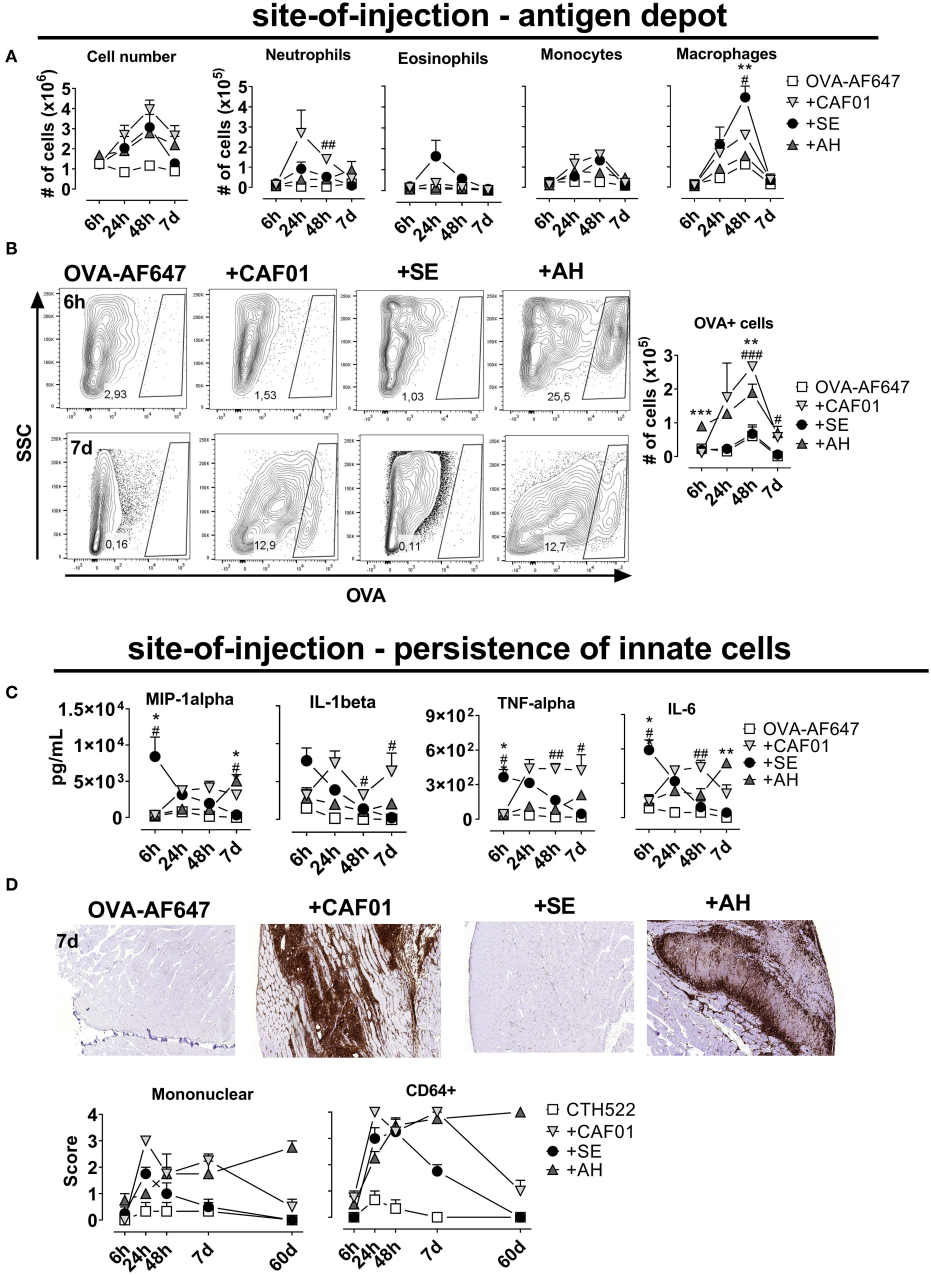
Persistence of antigen at the site of injection is influenced by adjuvant system. Mice were injected into the quadriceps muscle with ovalbumin (OVA) coupled to AF647 either alone or in the presence of the indicated adjuvant. **(A)** Total cell number (left panel) and the numbers of the indicated cell subsets (right panels) in the injected muscles at various time points after injection. **(B)** OVA-positive cells after injection. **(C)** Quantity of the indicated cytokines at various time points after injection. Mouse groups consisted of 12 mice per group with 3 mice per group sacrificed at each time point. Each point represents mean+ SEM. Statistically significant differences between the SE and AH groups are indicated by *, **, and ***, whilst significant differences between the SE and CAF01 groups are indicated by ^#, ##^, and ^###^ (One-way ANOVA with Tukey's correction for multiple group comparison, using the SE group as reference and significance levels of *p* < 0.05, *p* < 0.01, and *p* < 0.001, respectively). **(D)** Mice were vaccinated with CTH522 in the presence of the indicated adjuvants. The mice were scored by HE histology and immunofluorescent staining for mononuclear and inflammatory (CD64^+^) cells infiltrating the muscle at various time points after immunization. Representative plots display the 7 day time point. Three (antigen alone) or 8 mice (adjuvanted groups) were sacrificed at each time point.

To investigate antigen pharmacokinetics and uptake by innate cells, we performed injections of fluorescently labeled ovalbumin (OVA-AF647) alone or adjuvanted with CAF01, SE or AH. When administered intramuscularly, all adjuvants increased influx of cells to the muscle compared to antigen alone, with cell numbers peaking at 48 h after injection (Figure 2A). At 7 days post injection, cell numbers in the SE group had declined to levels similar to the antigen-alone group, whilst there were still 2-3-fold higher numbers in the other adjuvanted groups. We used the following gating strategy, modified from (15) to investigate the influx of immune cells into the injected muscle: neutrophils (CD11b^+^CD11c^−^Ly6G^high^), eosinophils (Ly6G^int^, F4/80^int^), macrophages (CD11b^+^, F4/80^high^), monocytes (CD11b^high^, F4/80-, CD11c^−^), DCs (CD11c^+^, CD11b^+/−^), and B cells (B220^+^) (Supplementary Figure 2). There was a rapid influx of neutrophils and eosinophils to the site of injection, whilst monocytes, macrophages and dendritic cells appeared later (Figure 2A, Supplementary Figure 2). When antigen was given in CAF01, higher numbers of neutrophils were recruited than with the other adjuvants (significant compared to the SE group at the 48-h time point, *p* = 0.001) (Figure 2A). In contrast, we observed more eosinophils and macrophages when OVA was injected with SE compared to CAF01 and AH (Figure 2A). Examining cells in the injected muscle that had acquired antigen (OVA^+^), we found similar numbers in the group injected with antigen in SE and the antigen-alone group (Figure 2B), whilst numbers were higher in the CAF01 and AH groups (significant at the 48-h time point, *p* = 0.007–0.009).

Adjuvants increase the levels of pro-inflammatory cytokines at the site of injection and we investigated if the depot-effect would correlate with persistence of cytokine responses. We measured a panel of cytokines (MIP-1a, Il-1B, TNF-a, IL-6, KC/GRO, IL-10, IL-12p70, and IL-5) in the muscle supernatant at various time points after injection using electrochemiluminiscence (MSD). When antigen was formulated in SE, the highest cytokine levels were observed already 6 h after administration and the cytokine response then decreased to reach levels comparable to the antigen-alone group at 7 days post administration (Figure 2C, Supplementary Figure 1). For the CAF01 and AH groups, responses were first detected at 24 h after administration and remained relatively constant throughout the study, although cytokine levels in the AH group remained relatively low until day 7. We measured how the different adjuvants influenced persistence of innate immune cells at the site of injection, by injecting antigen (CTH522) alone or formulated with the three adjuvants and performing HE staining at different time points after administration. The injected muscles were scored for presence of mononuclear cells and stained by anti-CD64 as a marker for innate cell infiltration. All adjuvants increased mono- and multinuclear cell numbers (Figure 2D), but at the 7-days-time-point muscles injected with SE scored negative for the presence of these cells, whilst they were still observed in the CAF01 and AH groups. Similar kinetics were found for CD64 expression, which was higher in tissue samples from the CAF01 and AH groups compared with the SE group at 7 days post administration. Notably, muscles injected with either CAF01 or AH, had CD64^+^ cells present even 60 days post administration. Thus, in contrast to SE, CAF01, and AH facilitated vaccine depot formation with persistent infiltration of innate immune cells.

### Depot Formation Is Associated With Reduced Antigen Drainage to Proximal Lymph Nodes

**Figure 3:**
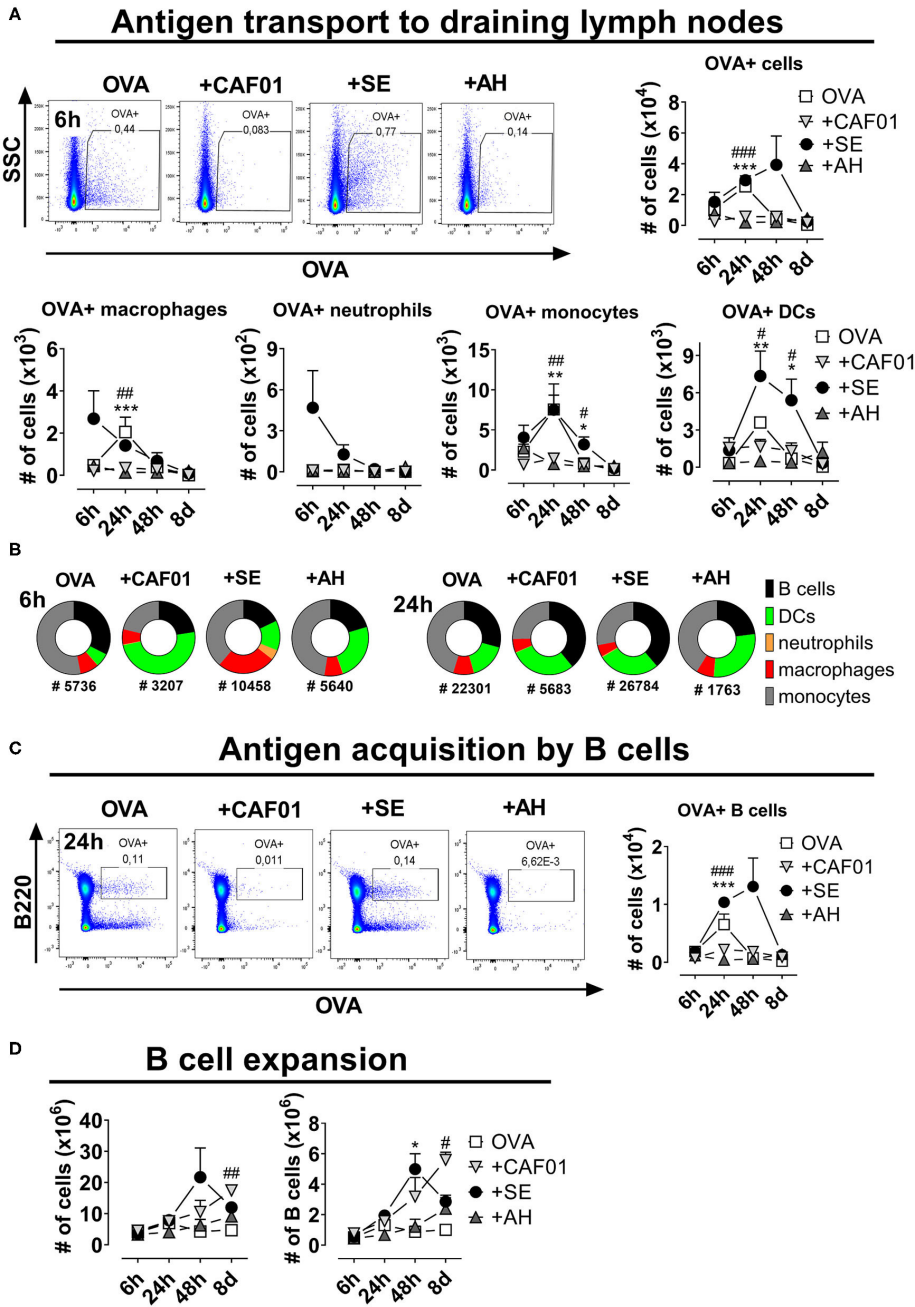
Reduced antigen drainage to proximal lymph nodes in the presence of depot-inducing adjuvants. Mice were injected subcutaneously at the base of tail with ovalbumin (OVA) coupled to AF647 either alone or in the presence of the indicated adjuvant and the draining inguinal lymph nodes were collected at various time points thereafter. **(A)** Cells binding to OVA in the draining lymph nodes. Representative plots display the 6-h-time-point (upper panels). Lower panels display the numbers of cells within the indicated subsets binding to OVA. **(B)** Fraction of cells within the indicated subsets binding to OVA at 6 and 24 h post administration. The total numbers of OVA^+^ cells (surface-adsorbed or internalized) are displayed below the pies. **(C)** Representative plots of B cells binding to OVA at 24 h post injection (left panel) and summarized for the different time points (right panel). **(D)** The total cell number (left panel) and the percentage and numbers of B220^+^ B cells (right panels) at the indicated time points after injection. Each group consisted of 3 (naïve and antigen alone) or 4 (for each adjuvant) mice evaluated at each time point. Each point represents mean+ SEM. Statistically significant differences between the SE and AH groups are indicated by *, **, and *** whilst significant differences between the SE and CAF01 groups are indicated by ^#, ##^, and ^###^ (One-way ANOVA with Tukey's correction for multiple group comparison, using the SE group as reference and significance levels of *p* < 0.05, *p* < 0.01, and *p* < 0.001, respectively).

B cell priming predominantly occurs in the follicle of the draining LN, which requires active or passive transport of antigen to this site. To investigate influence of the different adjuvants on antigen transport to the draining LN, we characterized LN cell association with fluorescently labeled antigen (OVA-AF647) injected s.c. Overall fewer antigen^+^ cells were found in draining LNs of mice having received CAF01 or AH adjuvanted vaccines compared to in the SE group or in mice immunized with antigen alone (Figure 3A). In the SE group a significant number of CD11b^+^F4/80^+^ macrophages were antigen^+^ already 6 h after administration (Figure 3A, Supplementary Figure 3). OVA^+^ Ly6G^+^ neutrophils, were also present in the group adjuvanted with SE, as reported previously for MF59^TM^ (15). At the 24-h-time point, increased numbers of OVA^+^ CD11c^+^ DCs appeared in the SE and antigen-alone groups. The cell type distribution of antigen^+^ cells varied between the different vaccine groups (Figure 3B). Thus, at 6 h post administration, antigen^+^ cells were mainly found amongst monocytes in the antigen alone group and DCs in the CAF01-adjuvanted group. In contrast, a substantial fraction of antigen^+^ cells in mice having received SE were macrophages. After 24 h the cell type distribution amongst antigen^+^ cells in the DLN was more similar between the different adjuvanted groups. Whilst few DLN B cells had bound OVA at 6 h post administration, high numbers of antigen^+^ B cells were found in the DLN after 24 h (Figure 3C). Up to 1 × 10^4^ antigen^+^ B cells were observed in the draining LNs of mice in the OVA-alone and the OVA in SE groups, whereas 10-50-fold lower numbers were detected in mice immunized with OVA in CAF01 or AH (significant, *p* = 0.0001). Similar results were observed when injecting the OVA-adjuvant combinations intramuscularly (data not shown). Activation of B cells in the draining LN following vaccination leads to expansion of these cells. All the tested adjuvants increased overall and B cell numbers in the draining LN (Figure 3D). The expansion occurred with different kinetics though, with the highest cell numbers found in the SE group at 48 h post injection, and after 8 days in the CAF01 and AH groups. Overall, administering antigen in the depot-inducing adjuvants CAF01 and AH led to reduced antigen drainage to proximal LNs and delayed B cell expansion, compared to when administered in SE adjuvant.

### Germinal Center Formation Is Delayed When Using Depot-Forming Adjuvants

**Figure 4:**
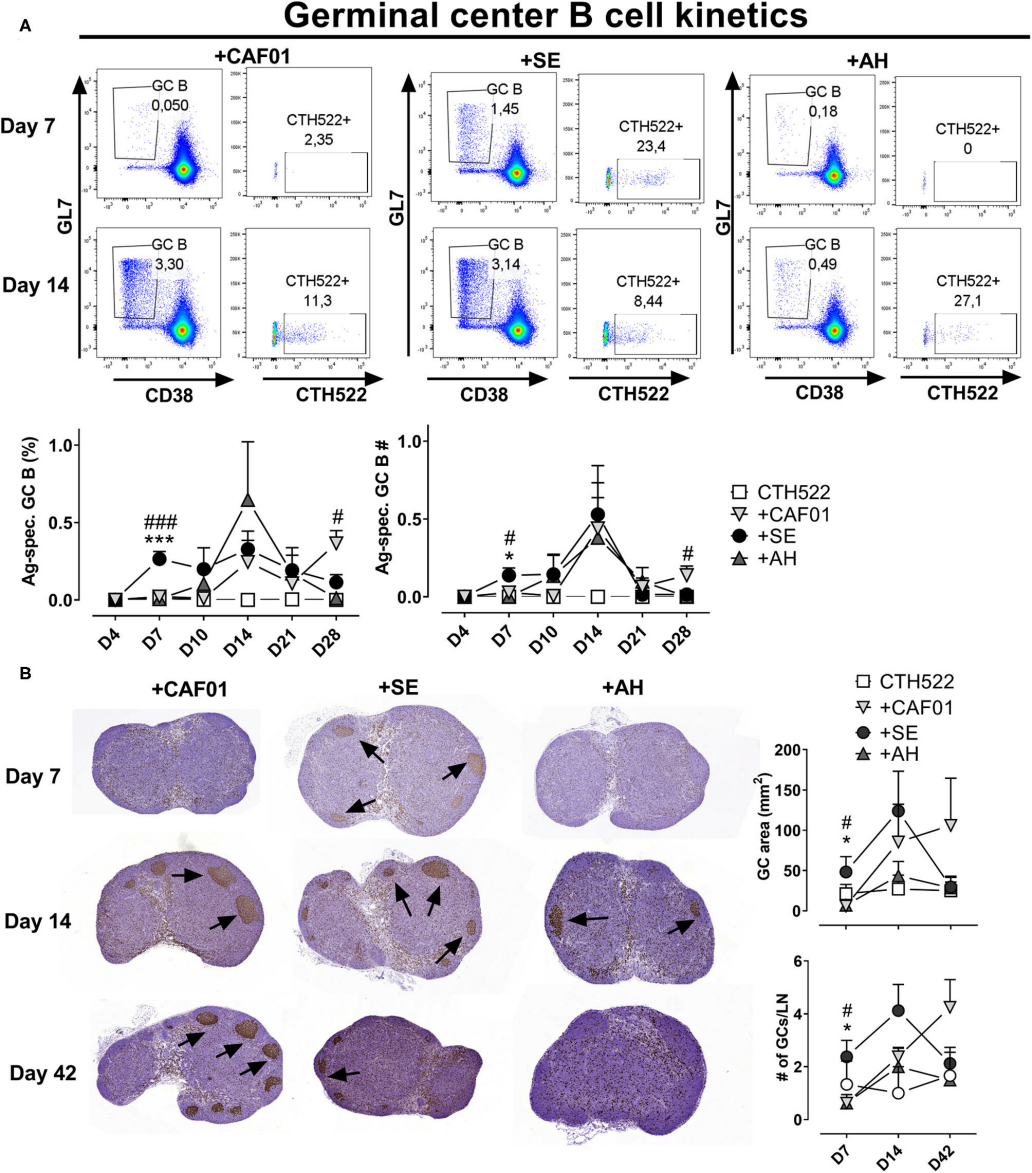
Adjuvants differentially influence germinal center kinetics. Mice were immunized subcutaneously with CTH522 protein antigen either alone or in the presence of the indicated adjuvant. **(A)** Representative plots of antigen-specific germinal center (GC) B cells (B220^+^CD38-GL7^+^ cells binding CTH522 coupled to AF488) in the draining inguinal lymph nodes at days 7 and 14 post immunization. Lower panels: percentages (of B220+) and numbers of antigen-specific germinal center B cells. Each group consisted of 2 (antigen alone) or 4 (antigen +adjuvants) mice. Data are representative of two independent experiments. **(B)** Draining lymph node GC areas (upper panel) and number of GCs as indicated by clusters of Ki67^+^ cells. Groups consisted of 3 (antigen alone) or 8 (antigen +adjuvants) mice. The experiment was performed once. Each point represents mean+ SEM. Statistically significant differences between the SE and AH groups are indicated by * and *** whilst significant differences between the SE and CAF01 groups are indicated by ^#^ and ^###^ (One-way ANOVA with Tukey's correction for multiple group comparison, using the SE group as reference and significance levels of *p* < 0.05 and *p* < 0.001, respectively).

Antibodies to protein antigens are predominantly produced from GC-derived plasma cells (11), although some of the antibody secreting cells can also be of extrafollicular origin (35). To test if the reduced amount of antigen delivered in draining LNs associated with administration in depot-forming adjuvants would lead to delayed GC formation, we compared the kinetics of GC B cell responses using antigen formulated in the depot- and non-depot-forming adjuvants. We used the CTH522 antigen for immunization and a fluorophore-labeled version of the antigen as a probe to evaluate antigen-specific GC B cell kinetics. Antigen alone did not induce any detectable antigen-specific GC B cells (Figure 4A). We also did not detect any GCs at the day 4 time point for any of the groups. At day 7 post immunization, a clear population of B220^+^CD38_lo_GL7^+^ GC B cells appeared in the SE group, of which on average 20% bound the labeled CTH522 probe. In contrast, GC B cells were not detected in the AH and CAF01 groups before at days 10 and 14, respectively (Figure 4A). The frequency of GC B cells declined at days 21 and day 28 in all groups, but was significantly higher in the CAF01 group compared to the SE group at day 28 (*p* = 0.020).

To confirm that the single cell stainings of GC B cells reflected germinal center formation, we evaluated Ki67 in H&E stained sections of draining LNs (Figure 4B). At 7 days post immunization, germinal centers indicated by clusters of Ki67^+^ cells could easily be observed in most of the mice that had received CTH522 antigen formulated in SE. In contrast, very few germinal centers were detected in the CAF01 and AH-adjuvanted groups and the germinal center area was significantly lower than in the SE group (*p* = 0.028–0.033). At 14 days post immunization, there were no significant differences in GC area between the adjuvanted groups, although there was a tendency toward lower GC responses in the AH group. At day 42 post immunization, similar high levels of GCs could still be detected in some of the mice that had received CAF01-adjuvanted vaccine, whilst GC levels were lower in the SE (not significant, *p* = 0.09) and AH groups. Thus, GCs appeared earlier when antigen was administered in SE than when formulated with CAF01 or AH.

### Removal of the Injection Site Abrogates Antibody Responses When Antigen Is Formulated in Depot-Inducing Adjuvants

**Figure 5:**
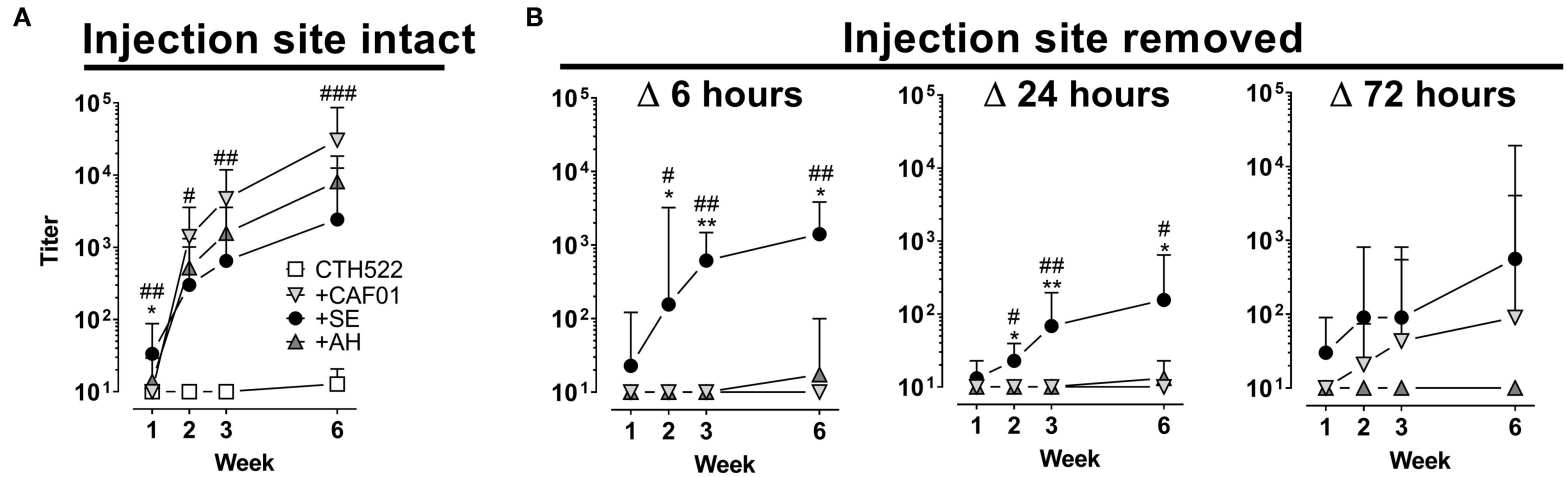
Removal of the injection site impairs antibody responses when antigen is formulated in depot-inducing adjuvants. Mice were immunized intradermally (i.d.) with CTH522 protein antigen either alone or in the presence of the indicated adjuvant. At various time points after injection, mice were anesthetized and the injection site either surgically removed or left intact. **(A,B)** Antigen-specific IgG antibody responses were measured at the indicated time points. Each vaccine group consisted of three to four mice and data are displayed as geometric mean+95% CI. Data in which the injection site was left intact (left panels) were compiled from three experiments. Statistically significant differences between the SE and AH groups are indicated by * and ** whilst significant differences between the SE and CAF01 groups are indicated by ^#^, ^##^, and ^###^ (One-way ANOVA with Tukey's correction for multiple group comparison, using the SE group as reference and significance levels of *p* < 0.05, *p* < 0.01, and *p* < 0.001 respectively).

Recent studies have questioned the role of the AH-induced antigen depot in elicitation of immune responses (8). We therefore investigated how removing the vaccine depot would affect antibody responses for depot vs. non-depot inducing adjuvants. For the surgery to be minimally invasive, we used intradermal (i.d.) immunizations. Injecting the CTH522 antigen in the various adjuvants confirmed that this administration route gave similar antibody kinetic profiles for the tested adjuvants as after s.c. immunization (Figure 5A). Removal of the vaccine depot at 6 and 24 h post administration completely abrogated antibody responses in mice having received AH and CAF01 adjuvanted vaccine (Figure 5B). In contrast, for the SE group, antibody responses were observed despite injection site ablation. Even when removing the antigen depot at 72 h post administration, antibody responses were still reduced in the CAF01 group and completely abrogated in the AH group. These data confirm that most antigen formulated in AH and CAF01 remains at the site of injection, at least for the first 24–72 h post i.d. administration, and it is possible that this depot effect may be responsible for the slower onset of GC formation and antibody responses.

## Discussion

Germinal centers (GCs) form in secondary lymphoid organs in response to immunization with T-cell-dependent antigens. Upon acquiring antigen an immune cascade is elicited where responding B cells form GCs to undergo proliferation, affinity maturation and class-switching and obtain one of two productive fates; memory B cells or plasma cells (36). During acute infections, microorganisms may replicate for weeks, thus providing continuous supply of antigen that may enter and sustain germinal center reactions. Soluble protein from subunit vaccines can be detected in the draining LNs already a few hours post immunization and is cleared much more rapidly, with intact antigen being non-detectable at 24–72 h later (11, 37). Mature naïve B cells are not found in muscle tissue, a common site for vaccine injection, but are circulating between follicles of secondary lymphoid organs. Initiation of T-cell dependent antibody responses requires that B cells in draining LN follicles encounter the injected cognate antigen (13). Inspired by studies indicating that particularly depot-inducing adjuvants elicit poor antibody responses early after a priming immunization (9, 27, 38), we hypothesized that slow release of vaccine from the site of injection, when antigen is formulated in depot-forming adjuvants, would lead to a delay in elicitation of B cell responses. It was previously demonstrated that more antigen-binding cells can be observed in the draining LNs of mice vaccinated with antigen formulated in MF59^TM^ or AS03 compared to in AH (15, 25). We confirmed these data and found that formulation in CAF01 also led to significantly reduced numbers of antigen-positive cells in draining LNs compared to when antigen was given alone or in an MF59^TM^-like SE adjuvant. Thus, whilst SE facilitated delivery to follicular B cells in the proximal LN (Figure 3), most antigen formulated in CAF01 or AH remained at the site of injection, which correlated with a delay in GC formation and antibody responses. In a recent study using osmotic pumps to facilitate slow delivery immunization, GC responses were also delayed compared to a bolus vaccine (23). Overall this suggests that antigen delivered slowly (e.g., by osmotic pumps or depot-inducing adjuvants, limits the amounts of antigen available for naïve B cells in proximal LNs, which reduces the chances of early B cell activation and formation of GCs).

The tested adjuvants induced marked differences in the cell types recruited to the site of injection after i.m. imunization. SE induced the highest influx of eosinophils and F4/80+ macrophages, whilst CAF01 induced the highest influx of neutrophils. It has been proposed that Trehalose 6,6′-dimycolate (TDM), of which the immunostimulatory component of CAF01 (TDB) is a synthetic analog, can function as a neutrophil chemoattractant (39). Notably, although AH induced the lowest influx of Ly6G^+^ neutrophils and F4/80^+^ macrophages of the adjuvants when measured by flow cytometry, immunohistology revealed many CD64^+^ cells in the injected muscle, which could be macrophages or inflammatory monocytes. In a previous study, AH was also demonstrated to attract F4/80^+^ macrophages to the site of injection (40). However, in the current study more F4/80^+^ macrophages were detected in the other adjuvanted groups, suggesting that at least relatively to SE and CAF01, AH induces little influx of F4/80^+^ macrophages. The infiltrating innate cells persisted at the site of injection long-term post injection of CAF01 and AH, whilst there was a more rapid decline in these cells in the SE group. We also observed striking differences in the distribution of immune cells acquiring antigen in the draining LN amongst the different adjuvanted groups. Despite CAF01 recruiting neutrophils to the site of injection, few antigen-positive neutrophils were seen in the draining LN. In contrast, SE induced limited recruitment of neutrophils to the site of injection, yet facilitated a rapid increase in antigen^+^ neutrophils in the draining LN 6 h post injection, as described previously (15). It is possible that SE adjuvant facilitates neutrophil-mediated transport of antigen from the site of injection to the draining LN, however neutrophil depletion experiments demonstrated that these cells are redundant for the ability of MF59^TM^ to promote antibody responses (15).

**Figure 6:**
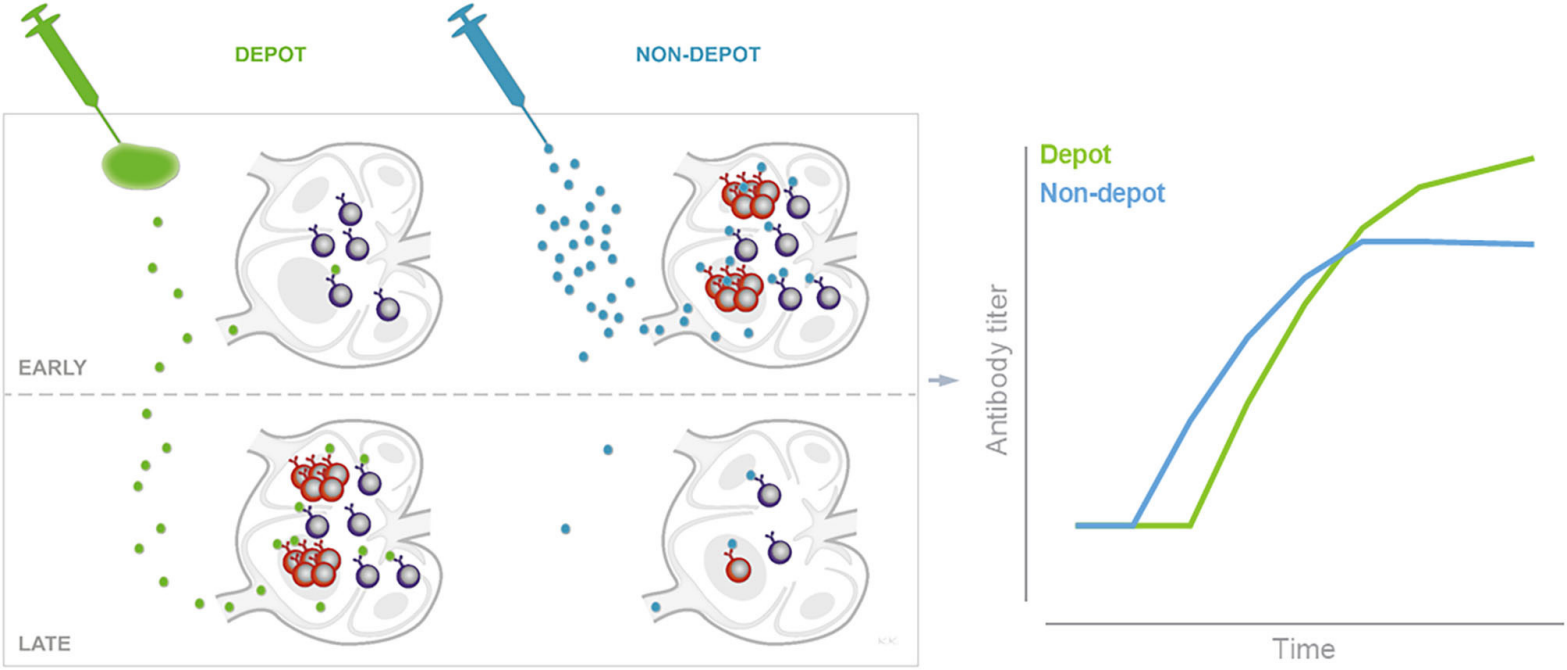
Schematic of depot formation affecting induction and persistence of germinal centers. Depot-forming adjuvants sequester antigen at the site of injection. Early after immunization this leads to reduced B cell activation and GC formation in the draining LNs, compared to when antigen is given in an adjuvant which does not form an antigen depot. The slow release of antigen from the depot may help sustain germinal centers to promote a higher magnitude long-term antibody response as illustrated by data adapted from Figure 1 (right panel).

Sustained antigen delivery [e.g., *via* osmotic pumps or repeated injections can greatly increase the magnitude of antibody responses (17, 23)]. Theoretically, adjuvants forming antigen depots hold the promise to also promote sustained delivery of antigen. In the present studies the depot-forming adjuvants CAF01 and AH, despite initially delaying germinal center formation, promoted antibody responses, which were of higher magnitude than the non-depot forming SE adjuvant later on (Figures 1, 5 and summarized in Figure 6). Although we found that adjuvants differentially influenced antigen drainage to proximal LNs and that this correlated with the kinetics of germinal center formation and antibody responses, it is difficult to ascertain that these events are directly connected. Thus, it is possible that the different immune profile stimulated by the non-depot-inducing adjuvant SE compared to CAF01/AH is responsible for the different kinetics of antibody responses, rather than the differences in drainage patters. Along these lines, Hutchison et al. showed that injection site ablation as early as 2 h post injection had no impact on antibody responses to Alum-adjuvanted vaccine, suggesting little if any role of an antigen depot (8). Whilst this study used ear ablation for injection site removal, we used another more clinically relevant i.d. route. Although it would have been relevant to study injection site removal after s.c. immunization to directly compare with studies of antibody response kinetics (Figure 1), we chose to perform the surgery after i.d. immunization to make it less invasive. However, we found that although different routes of immunization may influence kinetics of the immune response, similar kinetics of antibody responses were observed after s.c. and i.d. administration (Figures 1, 5), which included the pattern that SE facilitated a rapid IgG antibody response, whilst AH and CAF01 gave delayed antibody responses, ultimately reaching higher magnitudes. Removing the injection site within 24 h post administration of CTH522 in AH or CAF01 completely abrogated antibody responses (Figure 5), suggesting that most antigen formulated in AH or CAF01 remained at the site of injection for this period of time. The discrepancy between the study by Hutchison et al. and ours may be due to the differences in route (8) or antigens used (CTH522 vs. OVA). Antigens may adsorb to cationic liposomes and AH mainly by electrostatic, ligand exchange and hydrophobic interactions (41, 42). Thus, the degree of antigen depot and kinetics of antigen drainage are expected to be different for antigens with other physicochemical characteristics than those used in the present study [e.g., positively charged proteins (The antigens used here were 43–50 kDa proteins with pIs of 4.5–5.2)] (43, 44). However, although many antigens rapidly elute from AH after injection (45–47), we found high numbers of OVA^+^ cells at the injection site, even 7 days post administration of OVA in AH (Figure 2).

In the present study the depot-inducing adjuvants (CAF01 and AH) elicited IgG responses of similar kinetics and, for both, IgG titers continued to rise until the last time point 6 weeks post immunization. In a recent clinical trial evaluating a *Chlamydia trachomatis* vaccine, CAF01 elicited significantly higher IgG antibody responses than AH following a prime-boost regimen with the CTH522 antigen (32). Notably in that study, the IgG responses were boosted to much higher levels by the second booster (at 16 weeks) compared to after the first booster (at 4 weeks). It is generally believed that booster immunizations are most effective when administered after antibody responses have peaked (48). Given the data of the current study demonstrating that antibody responses mature slowly, with the highest IgG titers not observed before the end of the study (week 6), it seems possible that higher antibody responses would be obtained if the first booster vaccine was given later than 4 weeks after priming. For certain vaccines, limited spacing between immunizations has even been suggested to promote reduced responses to later administered booster doses [e.g., for the serogroup C meningococcal (Men-C)-conjugate vaccine], a single dose rather than a two-dose regime (spaced 1 month apart) primed for higher antibody responses to later boosting at 12 months of age (49). Thus, kinetics of antibody responses after prime immunization, and how these are modulated by adjuvants, warrants further study, in particular for designing prime-boost regimes.

We demonstrated that the antibody responses mature with different kinetics dependent on the adjuvant used. Specifically, two adjuvants (CAF01 and AH) which formed antigen depots at the site of injection induced delayed germinal center formation but promoted higher antibody responses than a non-depot-inducing adjuvant (SE) after a single immunization. These results are important for several reasons. First, considering adjuvant-dependent immune response kinetics is important in adjuvant comparison studies; second, the optimal time point for booster immunization, which is after contraction of germinal centers (48), may be dependent on the adjuvant used and third, by carefully considering antigen and adjuvant compatibility, and through rationally designing adjuvants to release antigen in a controlled manner, it may be possible to further promote antibody responses to protect against diseases where high magnitude somatically mutated antibodies are required. Understanding the immune kinetics controlled by the adjuvant is therefore of highest importance when comparing vaccines both pre-clinically and clinically.

## Data Availability Statement

The raw data supporting the conclusions of this article will be made available by the authors, without undue reservation.

## Ethics Statement

Mouse studies were conducted in accordance with the regulations set forth by the National Danish animal protection committee and in accordance with European Community Directive 86/609. The experiments performed have been approved by the Governmental Danish Animal Experiments Inspectorate under licenses 2014-15-2934-01065 and 2017-15-0201-01363.

## Author Contributions

GP, PA, and DC designed research and wrote the paper. GP and KW performed experiments. GP and DC analyzed data. All authors contributed to the article and approved the submitted version.

## Conflict of Interest

PA and DC are co-inventors on patents on the cationic adjuvant formulations (CAF). All rights have been turned over to Statens Serum Institut, which is a non-profit government research facility. The remaining authors declare that the research was conducted in the absence of any commercial or financial relationships that could be construed as a potential conflict of interest.
