# Supplementary material for: Vaccine Adjuvants Differentially Affect Kinetics of Antibody and Germinal Center Responses
Source: Front Immunol. 2020 Sep 23;11:579761. doi: 10.3389/fimmu.2020.579761 (PMC7538648; doi:10.3389/fimmu.2020.579761)
Supplement: Supplementary file 1 [file Data_Sheet_1.DOCX]

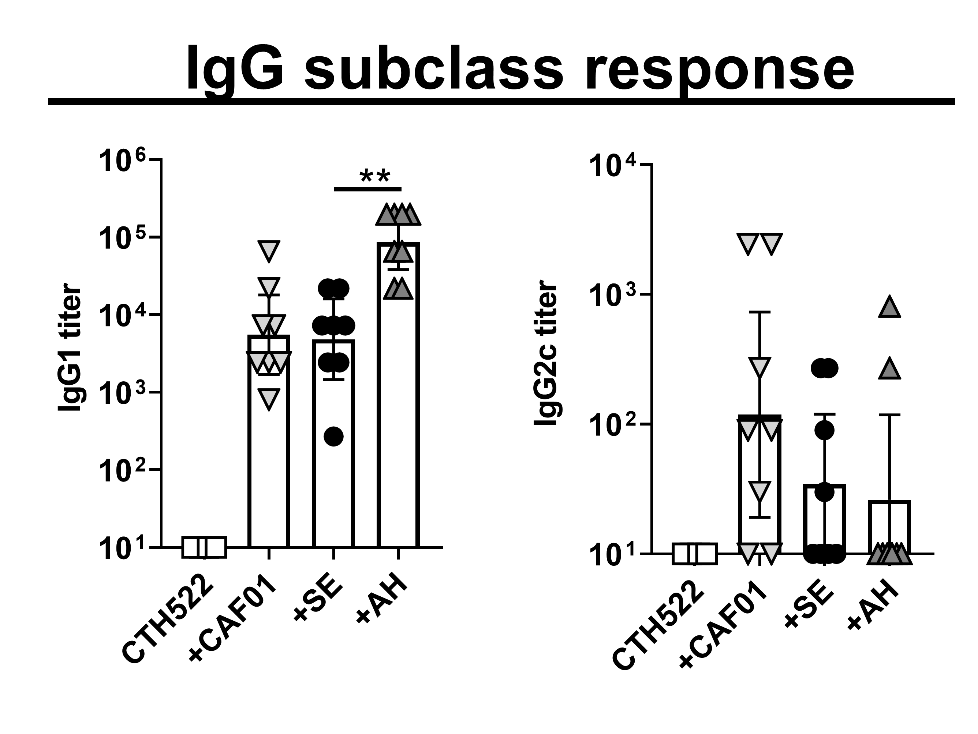


**sFig 1 IgG subclass responses after a single immunization**

Mice were vaccinated subcutaneously with 5µg of the recombinant protein antigen CTH522 either alone or in the presence of CAF01, SE (squalene emulsion) or AH (aluminium hydroxide). Antigen-specific IgG antibody responses were measured at 42 days post a single immunization. Data show titers expressed as geometric mean+95%. Statistically significant difference between the SE and AH group is indicated by ** Kruskal Wallis test, using the SE group as reference and significance levels of p<0.01.

**
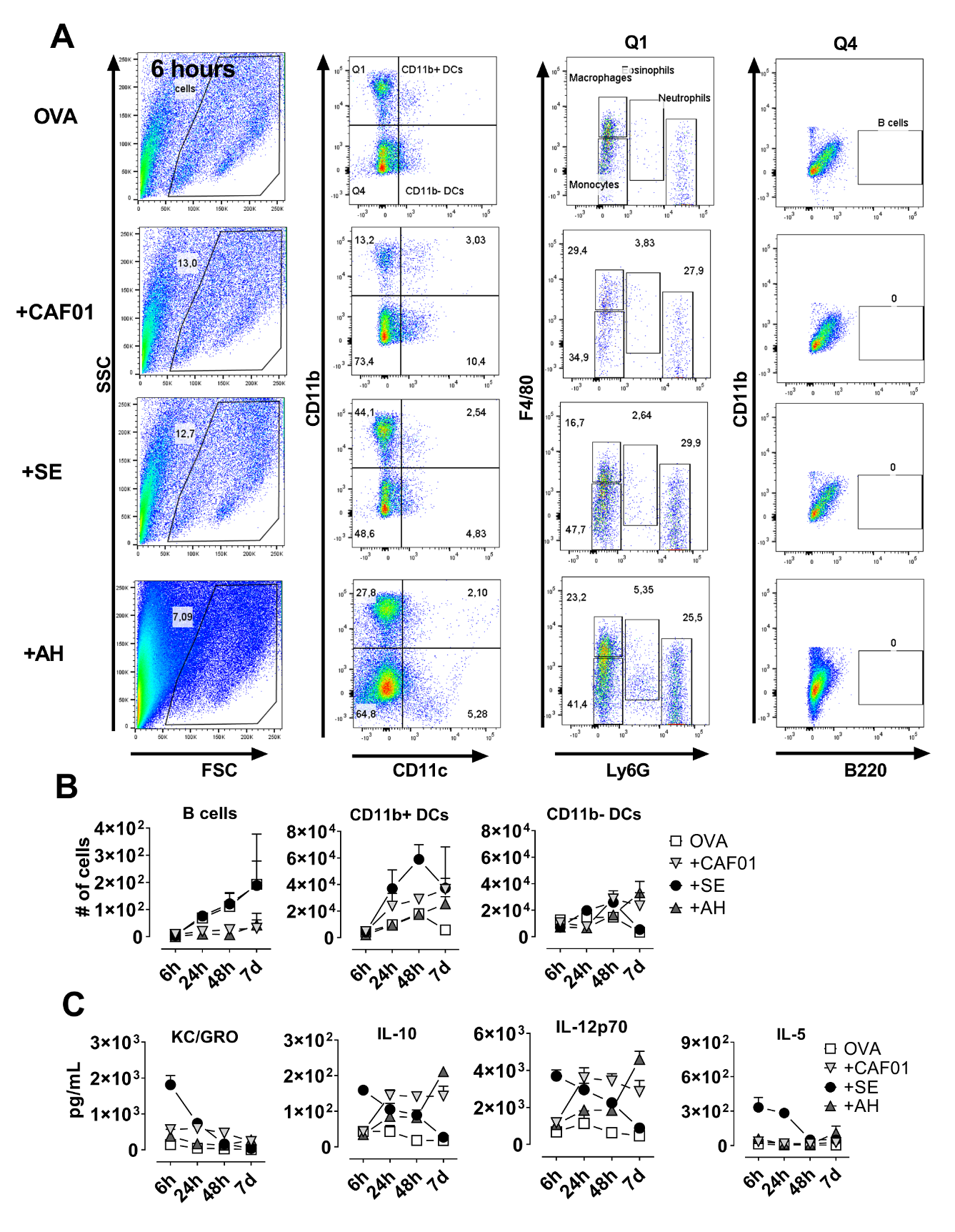
**

**sFig 2 Antigen depot at the site of injection**

Mice were injected into the quadriceps muscle with ovalbumin (OVA) coupled to AF647 either alone or in the presence of the indicated adjuvant. A) Representative gating for immune cells in the injected muscle. B) Numbers of the indicated cell subsets. C) Quantity of the indicated cytokines at various time points after injection. Mouse groups consisted of 12 mice per group with 3 mice per group sacrificed at each time point. Each point represents mean+ SEM.

**
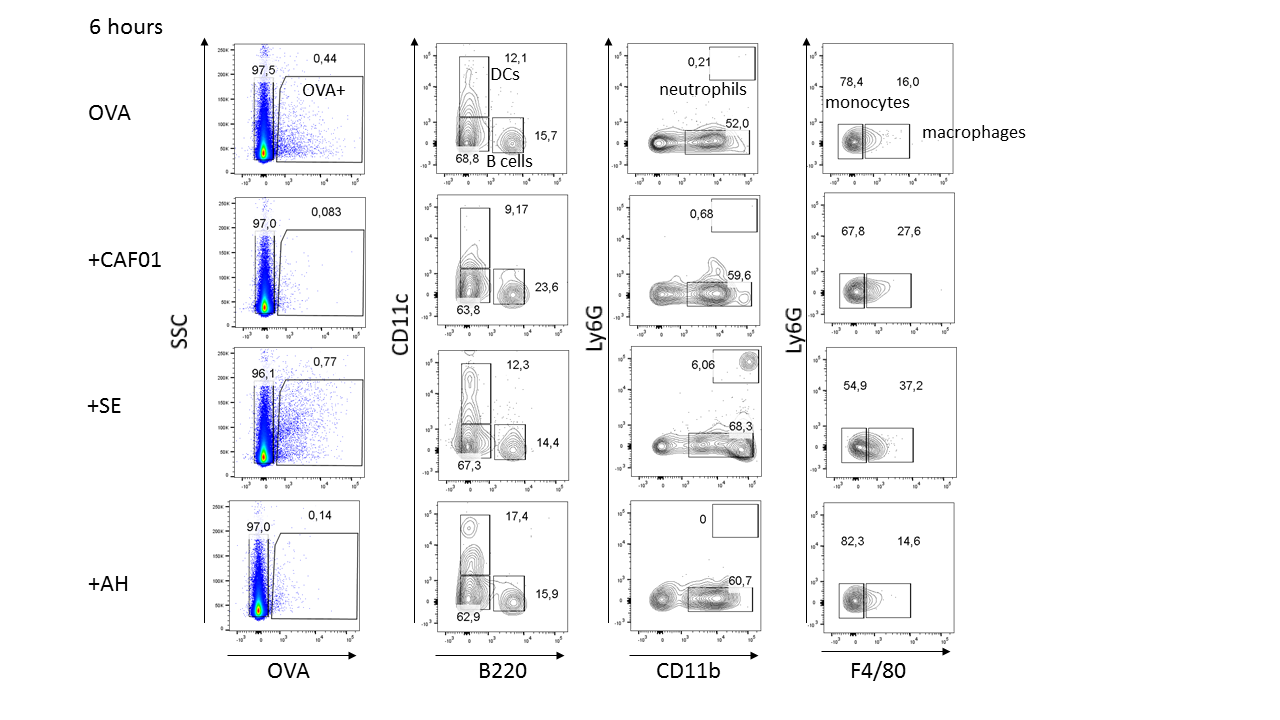
sFig 3 Representative gating of OVA-binding cells in the draining lymph node**

Mice were injected subcutaneously with ovalbumin (OVA) coupled to AF647 either alone or in the presence of the indicated adjuvant. Representative gating for immune cells in the draining lymph node at 6 hours post injection is displayed.
